# Solution-Based Synthesis of Few-Layer WS_2_ Large Area Continuous Films for Electronic Applications

**DOI:** 10.1038/s41598-020-58694-0

**Published:** 2020-02-03

**Authors:** Omar A. Abbas, Ioannis Zeimpekis, He Wang, Adam H. Lewis, Neil P. Sessions, Martin Ebert, Nikolaos Aspiotis, Chung-Che Huang, Daniel Hewak, Sakellaris Mailis, Pier Sazio

**Affiliations:** 1 Optoelectronics Research Centre, University of Southampton, Southampton, SO17 1BJ United Kingdom; 2 National Centre for Advanced Tribology, University of Southampton, Southampton, SO17 1BJ United Kingdom; 3 School of Electronics and Computer Science, University of Southampton, Southampton, SO17 1BJ United Kingdom; 4 Present Address: Skolkovo Institute of Science and Technology Novaya St., 100, Skolkovo, 143025 Russian Federation

**Keywords:** Two-dimensional materials, Electronic devices, Materials for devices, Electronic properties and materials, Synthesis and processing

## Abstract

Unlike MoS_2_ ultra-thin films, where solution-based single source precursor synthesis for electronic applications has been widely studied, growing uniform and large area few-layer WS_2_ films using this approach has been more challenging. Here, we report a method for growth of few-layer WS_2_ that results in continuous and uniform films over centimetre scale. The method is based on the thermolysis of spin coated ammonium tetrathiotungstate ((NH_4_)_2_WS_4_) films by two-step high temperature annealing without additional sulphurization. This facile and scalable growth method solves previously encountered film uniformity issues. Atomic force microscopy (AFM) and transmission electron microscopy (TEM) were used to confirm the few-layer nature of WS_2_ films. Raman and X-Ray photoelectron spectroscopy (XPS) revealed that the synthesized few-layer WS_2_ films are highly crystalline and stoichiometric. Finally, WS_2_ films as-deposited on SiO_2_/Si substrates were used to fabricate a backgated Field Effect Transistor (FET) device for the first time using this precursor to demonstrate the electronic functionality of the material and further validate the method.

## Introduction

2D transition metal dichalcogenides (TMD) have emerged as promising low dimensional semiconductor materials^1^ due to their exceptional electrical, optical and mechanical properties^2^. Among the TMD family, MoS_2_ has been the first and most investigated member because of its excellent properties such as thickness dependent indirect to direct bandgap transition^3,4^, valley Hall effect^5^, high carrier mobility and on-off ratio^6^ that makes it suitable for a wide range of electronic applications. A significant number of scientific reports have addressed the synthesis routes of MoS_2_. The production methods reported in the literature include mechanical^6^ or liquid exfoliation^7^ as well as conventional atomic layer deposition (ALD)^8^ and chemical vapour deposition (CVD)^9^. These approaches however are still far from being commercially viable due to low yield and/or high costs.

Two significant factors that identify a successful route to commercialization of a material are its compatibility with existing fabrication methods and cost effectiveness. Solution-based synthesis is compatible with existing nanofabrication processes, is scalable at low cost and has already been shown to produce high quality MoS_2_ films using a single source precursor such as ammonium tetrathiomolybdate (NH_4_)_2_MoS_4_ through thermal decomposition for electronic devices applications^10^. Therefore, several groups have developed approaches for large area solution-based MoS_2_ synthesis via two-step thermolysis of (NH_4_)_2_MoS_4_ films coated in different ways such as dip, roll to roll and spin coating^11–18^. Spin coating of (NH_4_)_2_MoS_4_ solution in particular is highly preferable among other coating techniques due to its integration with current semiconductor technology and its ability to control the initial precursor film thickness through spinning speed as well as precursor solution concentration^13,17^. The main obstacle hindering this coating technique is the low wettability of precursor solutions that utilize common solvents such as dimethylformamide (DMF) and n-methylpyrrolidone (NMP), with commonly used substrates like SiO_2_/Si or sapphire. This leads to nonuniform precursor film formation associated with a high density of defects and de-wetted areas after spin coating. To overcome this issue, researchers developed different organic-precursor solution systems for spin coating to enhance the uniformity and controllability of the initial precursor film over large area and eliminate surface defects. These organic-precursor solutions systems are: DMF, n-butylamine and 2-aminoethanol^13^; ethylenediaminetetraacetic acid (EDTA) and dimethylsulfoxide (DMSO)^16^; and linear poly (ethylenimine), DMF and 2-aminoethanol^17^.

Similarly, WS_2_ is an important TMD material which shows comparable characteristics to MoS_2_ but can also offers higher photoluminescence (PL) efficiency, better electrical performance and ambipolar field effect behaviour^19–21^. Although WS_2_ has growth methods similar to MoS_2_ such as sulphurization of tungsten metal^22^ or tungsten oxides^23^, growth of large area and uniform WS_2_ ultra-thin films for electronic applications via thermal decomposition of ammonium tetrathiotungstate (NH_4_)_2_WS_4_ salt has not been demonstrated successfully. This is due to the difficulty that is associated with the formation of a thin uniform (NH_4_)_2_WS_4_ precursor layer as this salt has poor solubility in most of the common solvents as compared with (NH_4_)_2_MoS_4_^24^. Usage of these solvents in solution-based single source precursor deposition of WS_2_ has however been demonstrated for applications that do not require highly continuous WS_2_ films, such as surface enhancement Raman scattering (SERS) and creating a carrier injector layer for optoelectronic devices^25,26^. Another issue in solution-based single source precursor synthesis by thermal decomposition occurs when the second annealing step is relatively high (≤800 °C). In this case, sulphur from (NH_4_)_2_MoS_4_ and (NH_4_)_2_WS_4_ films evaporates readily and needs to be substituted by adding sulphur in the inert gas flow to preserve the stoichiometry and the quality of the MoS_2_ and WS_2_ films^11,13,25,26^.

Generally, there are three factors that need to be optimized to create a defect-free film from a liquid precursor by spin coating; the wettability of the precursor solution with the substrate, the solubility of precursor in the solvent system and the viscosity of the solution. Wettability can be significantly improved by oxygen plasma treatment of the substrate which promotes the hydrophilicity of the surface^13^. Choosing a solvent capable of achieving high solubility of the precursor helps to eliminate clusters and striation formation due to surface tension. Viscosity can be controlled by the choice of the solvents system and the concentration of the precursor, which affects the coverage of the film over the substrate and the final precursor film thickness^13,17^.

We have therefore optimized all three parameters to create uniform large area ultra-thin WS_2_ layers via a two-step thermal decomposition of (NH_4_)_2_WS_4_ spun-coated precursor solutions. A refined solvent recipe was formulated to improve the wettability and uniformity of the precursor film on the substrate. Additionally, by processing our samples in a “facing pair” manner during the high temperature second annealing step, the composition/stoichiometry of the films was preserved, thus eliminating the need for additional sulphurization. Optical microscopy was used to assess the uniformity and continuity of the precursor films while AFM and TEM evaluated the resulting WS_2_ film thickness and morphology. Raman spectroscopy supported the AFM and TEM results to identify the few-layer nature of the films and showed the effect of temperature on the crystallinity of the film. XPS spectroscopy revealed the stoichiometry of WS_2_ films when grown on different substrates. Finally, an FET device was fabricated using as-deposited WS_2_ film to further elucidate the potential electronic applications of these films.

## Results and Discussion

Electronic devices such as FETs require continuous and uniform films for the device layer to guarantee high electrical performance. In solution-based synthesis of semiconducting WS_2_ films, the main defects are pinholes and de-wetted areas over the substrate that occur in the precursor deposition step. In this work we have tackled these issues by refining the solvents system for the (NH_4_)_2_WS_4_.

There are simple solutions for wettability but because (NH_4_)_2_WS_4_ is weakly coordinated with most of the solvents^24^, the key challenge in making a solution-based uniform ultra-thin WS_2_ film is formulating a solution recipe that results in a high solubility of the precursor with the optimum viscosity. Based on this, we chose the most promising solvents reported previously for spin coating, namely (DMF)^27^, ethylene glycol^26^, (NMP)^12^ to investigate which one has the maximum solvation and coverage over the substrate. Preparation of the substrates and precursor solutions for spin coating is described in Materials and Methods.

**Figure 1:**
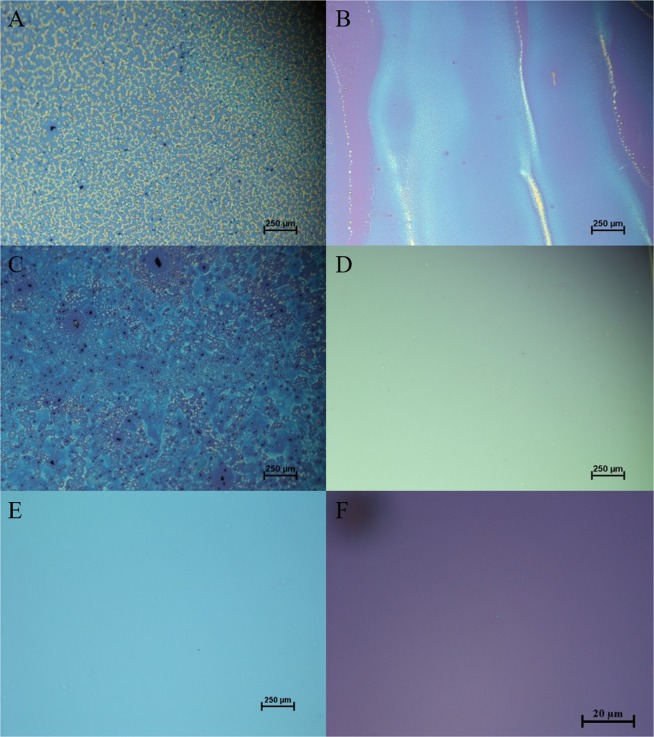
Optical microscope images of spin-coated precursor films prepared by dissolving 100 mM of (NH_4_)_2_WS_4_ in: (**A**) dimethylformamide (DMF), (**B**) ethylene glycol, (**C**) n-methylpyrrolidone (NMP) and (**D**) solvent system contains (3 mL NMP/2 mL n-butylamine/1 mL 2-aminoethanol of 6 mL total volume). (**E**,**F**) are optical microscope images of spin-coated precursor films prepared by dissolving 35 mM of (NH_4_)_2_WS_4_ in (3 mL NMP/2 mL n-butylamine/1 mL 2-aminoethanol of 6 mL total volume). All the solutions are spin coated at 6000 rpm for 1 min and prebaked at 140 °C for 1 min. Note that (**A–E**) images were taken using 5X objective while (**F**) image was taken using 100X objective.

Figure 1(A–C), shows the spin-coated precursor films where (NH_4_)_2_WS_4_ is dissolved in DMF, ethylene glycol and NMP solvents respectively (100 mM solution concentration), at 6000 rpm spinning speed. Unfortunately, none of the solvents successfully formed continuous and uniform (NH_4_)_2_WS_4_ films. The high concentration and therefore viscosity of the solutions should have facilitated the continuous film formation, opposite to what we observed here. Moreover, the de-wetted regions were significantly enlarged at 9000 rpm which was the peak of the spin coating speed (see Fig. S1). DMF was promising in terms of forming continuous precursor films with excellent surface coverage at 3000 and 6000 rpm speeds. Unfortunately, we identified high density of clusters that probably consist of insoluble WS_4_^−2^ anions. Ethylene glycol based film exhibited lower density of clusters as compared to DMF at 3000 rpm (see Fig. S1) but had more de-wetted regions, with the wettability deteriorating at moderate and high spin coating speeds, thus preventing the formation of a thin uniform layer. Unlike the previous solvents, NMP showed some more solvation without forming clusters at all spin coating speeds, indicating a better solvation of (NH_4_)_2_WS_4_ by NMP compared to DMF and ethylene glycol solvents. However, the high density of random pinholes assigned to insufficient wetting of the solution with the substrate.

It has been reported before that amine-based solvents could be linked with WS_4_^−2^ anions via hydrogen bonds leading to form a stable solution^28,29^. Additionally, it has been shown that n-butylamine and 2-aminoethanol solvents can stabilize the (NH_4_)_2_MoS_4_ and bind the solution to create uniform MoS_2_ precursor films by spin coating^13^. However, this (NH_4_)_2_MoS_4_ solvents recipe contains DMF rather than NMP which leads to non-uniform WS_2_ precursor layer formation when it was used for (NH_4_)_2_WS_4_ (see Fig. S2). Based on these facts, we reformulated the recipe of NMP by adding the solvents butylamine and 2-aminoethanol (see Materials and Methods). Moreover, to ensure good coverage and uniformity of the spin-coated precursor layer, we started with high concentration solution (100 mM), as is evident from Fig. 1(D) there was a significant improvement of the uniformity without any obvious de-wetted regions over a large area. The few apparent micron-sized defects originated from particles on the substrate. However, further reduction in precursor solution concentration was needed in order to achieve WS_2_ films with minimum thickness. Therefore, we used the same solvents recipe with the threshold concentration (35 mM) of (NH_4_)_2_WS_4_ that can produce large area and uniform precursor films as shown in Fig. 1(E–F) respectively. At lower concentrations than this (e.g. 10 or 20 mM), the density of WS_4_ anions in the solution were very low leading to formation of isolated micron-sized islands rather than a continuous film (see Fig. S3).

**Figure 2:**
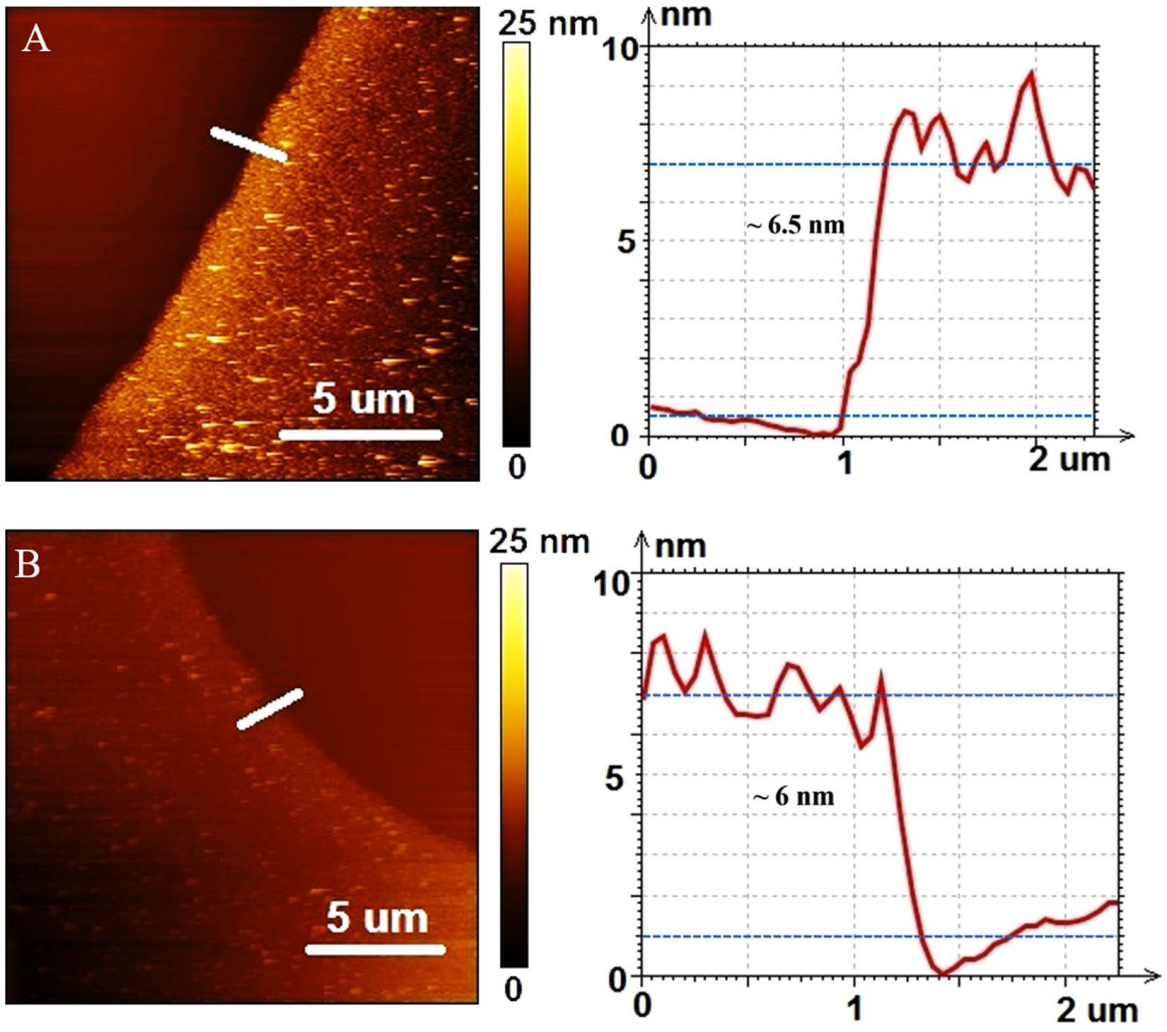
Atomic force microscopy (AFM) images of WS_2_ films grown on (**A**) SiO_2_/Si (**B**) sapphire.

After the second annealing step of WS_2_ films grown on SiO_2_/Si and sapphire substrates (with 35 mM precursor concentration), the topography and thickness of these samples were assessed by atomic force microscopy (AFM). The average thickness of the films for an area of edges was 6.5 ± 0.68 nm (0.68 nm is the root mean square roughness R_q_ of the film) for the SiO_2_/Si substrate and 6 ± 0.1 nm (0.1 nm is the root mean square roughness R_q_ of the film) for the sapphire as shown in Fig. 2. The higher roughness of the film grown on the SiO_2_/Si substrate is attributed to the amorphous nature of the substrate and to the increased surface roughness caused by the relatively long time exposed to oxygen plasma. As AFM measurements were taken at the edge of WS_2_ films where they are prone to edge effects from the spinning process, therefore, these AFM results represent the maximum thickness of WS_2_ films. Additionally, the AFM images confirm the continuity of the WS_2_ films with small grains that appear due to the nano-size crystals formed at the WS_2_ uniform film.

**Figure 3:**
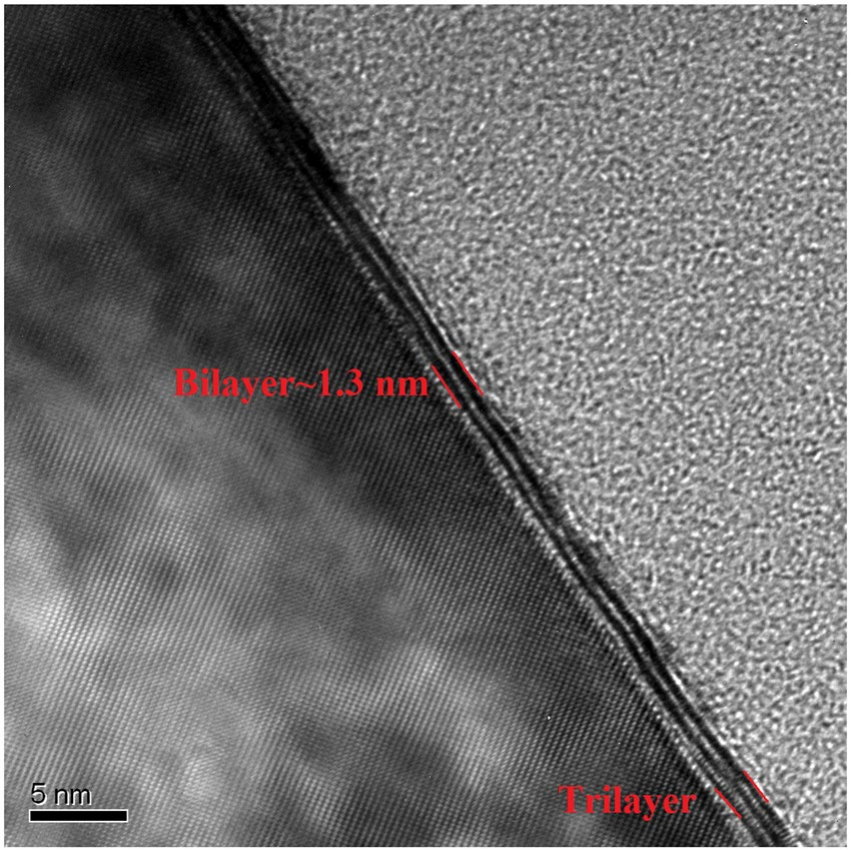
TEM image of few-layer WS_2_ films grown on sapphire substrate. The crystalline Al_2_O_3_ atomic lattice is clearly visible on the left hand side of the image. The WS_2_ film is viewed at a high angle where bilayer and trilayer regions are also highly visible and are indicated. The bright area on the right hand side is the protective carbon coating. The trilayer region also shows the WS_2_ atomic arrangement.

To evaluate the structure of the WS_2_ films at the central area of the samples, TEM was conducted for the film grown on a sapphire substrate. Figure 3 shows a TEM image of the WS_2_ film demonstrating a highly ordered layered structure. It is apparent from Fig. 3, the film is uniform and constituted by areas of 2 and 3 layers. The TEM image indicates that the single layer thickness of the WS_2_ film is 0.65 nm, in agreement with previously reported WS_2_ monolayer thickness^23^. At the bottom right of Fig. 3 and also in Fig. S4 arranged columns of atoms are clearly visible.

**Figure 4:**
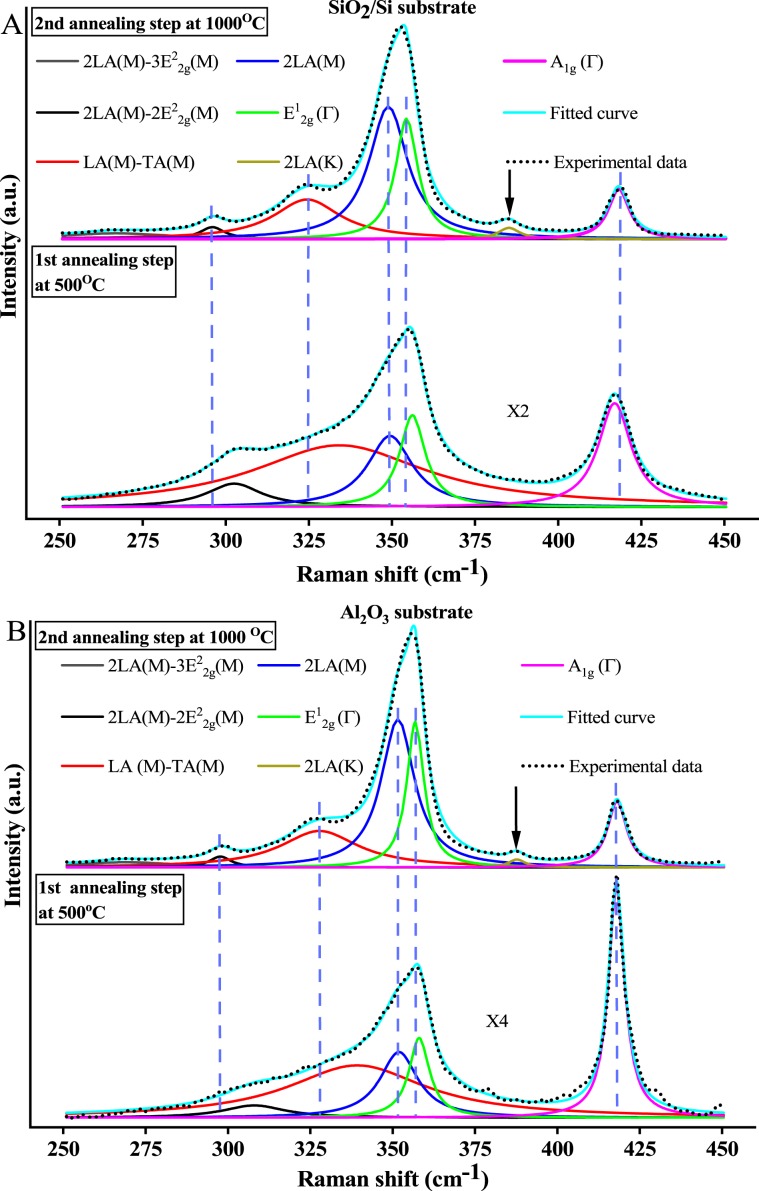
Raman spectra of WS_2_ films on (**A**) SiO_2_/Si and (**B**) Sapphire at the 500 °C and 1000 °C respectively.

Raman spectroscopy with a 532 nm excitation wavelength was performed to characterize the WS_2_ films on both SiO_2_/Si and sapphire substrates for each of the two-annealing steps (500 °C and 1000 °C). The consequence of using a 532 nm pump laser for Raman spectroscopy is an enrichment of the Raman spectra with second order peaks^30^. Multi-peak Lorentzian fitting is applied to deconvolute these peaks which helps to reveal any crystallinity changes between the two-annealing steps and to estimate the thickness of the films. As shown in Fig. 4 after the 1000 °C anneal, the Raman spectra intensity at the centre of the samples were enhanced by X2 for the SiO_2_/Si and X4 for the sample grown on sapphire. Moreover, all peaks become narrower after the second annealing step. The most dramatic change was with the peak labelled LA (M)-TA(M)^31^ where its FWHM was reduced from 63.4 cm^−1^ in the first annealing step to 24.4 cm^−1^ in the second annealing step for SiO_2_/Si and from 51 cm^−1^ to 28 cm^−1^ for sapphire. Additional peaks that correspond to 2LA (K) mode^32^ are also apparent at 385.2 cm^−1^ and 387.7 cm^−1^ for SiO_2_/Si and sapphire respectively, which did not exist after the first annealing step. The intensity enhancement in the Raman spectra, the reduction in full width half maximum (FWHM) of all Raman peaks as well as the prominence of additional second order peak (2LA (K)) highlight the significance of the second annealing step at high temperature (1000 °C) to promote the crystallinity of the WS_2_ films.

Furthermore, at 500 °C the intensity of the in-plane E^1^_2g_ (Γ) dominated the longitudinal 2LA (M) mode whereas at the 1000 °C the 2LA (M) peaks increased in intensity which almost overwhelmed the E^1^_2g_ (Γ) mode for all substrates. In contrast, the out-of-plane peak A_1g_ (Γ) decreased in intensity at the higher temperature. Consequently, the 2LA (M)/A_1g_ (Γ) intensity ratio increased dramatically from 0.685 at 500 °C to 2.64 at 1000 °C for the SiO_2_/Si and from 0.285 to 2.26 for the sapphire. Moreover, the Raman peak difference between the in-plane mode E^1^_2g_ (Γ) and out-of-plane mode A_1g_ (Γ) at 1000 °C is 63.8 cm^−1^ for the SiO_2_/Si substrate and 61.2 cm^−1^ for the sapphire substrate. Both the intensity ratio of 2LA (M)/A_1g_ (Γ) and Raman peaks difference (A_1g_ (Γ)- E^1^_2g_ (Γ)) indicate the few-layer nature of the measured WS_2_ films on both substrates similar to what has been reported in the literature^33,34^.

Interestingly, after the second annealing step all the peaks from E^1^_2g_ (Γ) to 2LA (M)-2E^2^_2g_ (M) are shifted to lower wavenumbers compared to their peak positions after the first annealing step at 500 °C. The only exception was the longitudinal acoustic mode 2LA (M), which did not shift after the two annealing steps for both substrates. As a result, the separation of the in-plane E^1^_2g_ (Γ) and the out-of-plane peak A_1g_ (Γ) Raman peaks after the first annealing step is smaller compared to after the high temperature annealing step (60.8 cm^−1^ for SiO_2_/Si and 59.9 cm^−1^ for sapphire). This is due to blue shifts that E^1^_2g_ (Γ) peaks experience in poor crystalline films as stated previously. However, the intensity ratio of 2LA (M)/A_1g_ (Γ) peaks is also low (0.685 for the SiO_2_/Si and 0.285 for the sapphire) for poor crystalline films. Thus, the layer number estimation of poor crystalline WS_2_ films (500 °C) using Raman spectra with 532 nm excitation wavelength might be not accurate. The reason behind this is the correlation between Raman peaks difference and the intensity ratio is not valid for poor crystalline WS_2_ films (500 °C) as opposed to the higher crystalline WS_2_ films (1000 °C) which show a clear correlation between Raman peak difference and intensity ratio when resonant excitation wavelength is used for Raman spectroscopy^33^.

**Figure 5:**
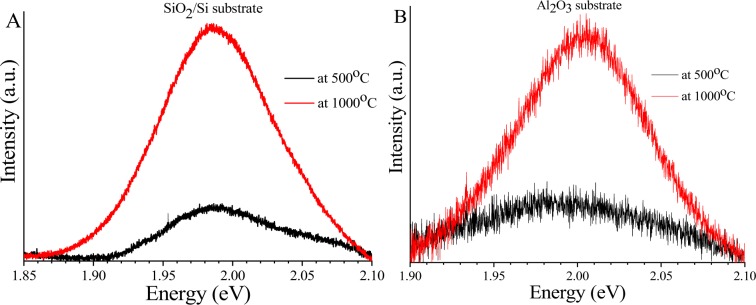
PL spectra of WS_2_ films on (**A**) SiO_2_/Si and (**B**) Sapphire at 500 °C and 1000 °C respectively.

The PL spectrum of WS_2_ films was investigated using the same excitation wavelength, power and objective parameters as the Raman measurements. As shown in Fig. 5 there is a significant enhancement of the photoluminescence (PL) signal after high temperature annealing for both substrates. The PL intensity enhancement is X4.5 for the SiO_2_/Si and X3.5 for the sapphire substrate and this enhancement is attributed to the improvement in film crystallinity. However, the PL peaks intensities are still weak, due to few-layer nature of films. For the SiO_2_/Si substrate, the PL peak of WS_2_ film is located at 1.984 eV, in agreement with earlier reports for few-layer WS_2_^31^, whereas the PL peak position of WS_2_ film grown on sapphire substrate is at 2 eV similar to what has been observed before for WS_2_ films grown on sapphire^35^. The trivial shift to lower energy in PL peak position of the WS_2_ films grown on SiO_2_/Si substrate compared to the films grown on sapphire might results from higher strain on the film deposited on SiO_2_/Si substrate^32^.

**Figure 6:**
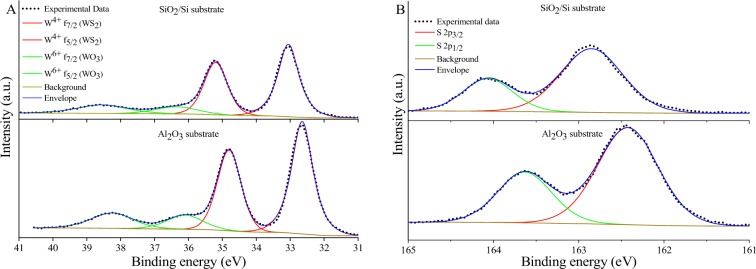
XPS spectra of WS_2_ films on SiO_2_/Si and sapphire substrates (**A**) W 4 f core-level and (**B**) S 2p core-level spectra.

The composition of the deposited WS_2_ films was investigated by high resolution XPS for both substrates where the W and S core levels were studied. The carbon peak in the C1s core level was used as a reference point and was at 284.8 eV for both substrates. The de-convoluted XPS spectra for W and S core levels are shown in Fig. 6(A,B) respectively for both substrates. For the WS_2_ film deposited on the SiO_2_/Si substrate, two doublets were pronounced in the W core level, the first doublet represents the W^4+^ f4_7/2_ at 33.05 eV and W^4+^ f4_5/2_ at 35.20 eV which is attributed to WS_2_ formation, with spin orbit splitting (W^4+^ f4_5/2_-W^4+^ f4_7/2_) of 2.15 eV and an area ratio of W^4+^ f4_5/2_/W^4+^ f4_7/2_ 0.73. The energy of these peaks corresponds to the 2 H phase of WS_2_^36^. The second doublet is located at 36.35 and 38.55 eV respectively and represents the W^6+^ f4_7/2_ and W^6+^ f4_5/2_ peaks that depicts the formation of WO_3_^37^. For sulphur, the S 2p_3/2_ and S 2p_1/2_ peaks are located at 162.85 and 164.06 eV respectively with spin orbit splitting (S 2p_1/2_-S 2p_3/2_) 1.21 eV and area ratio S 2p_1/2_/S 2p_3/2_ 0.4. This doublet corresponds to S^2−^ sulphur bonded in 2 H phase of WS_2_^36^. Fortunately, the absence of S_2_^2-^ ligands peaks, which corresponds to the presence of WS_3_ and oxidized sulphur species, is a good indicator that the film is stoichiometric for both substrates^37,38^. The peaks obtained from the WS_2_ films on the sapphire are almost identical to films grown on SiO_2_/Si. However, there is a shift to lower energies by 0.4 eV for all peaks assigned to WS_2_ in both W and S core levels and the oxide content is negligibly increased compared to films grown on SiO_2_/Si.

**Figure 7:**
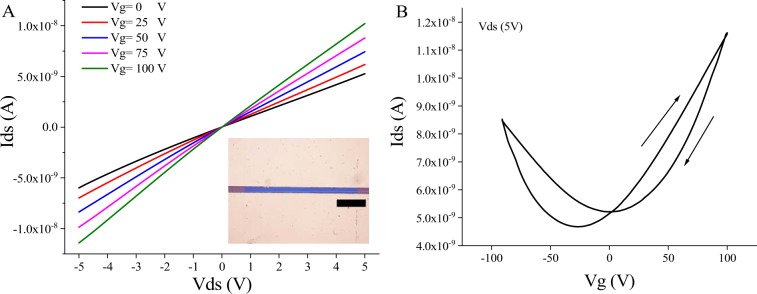
Electrical characteristics of back-gated WS_2_ FET (**A**) I_ds_-V_ds_ (inset: optical microscope image for the actual FET device, the scale is 50 μm). (**B**) Forward and backward sweep transfer characteristics.

To probe the electronic properties of the WS_2_ film, we fabricated a back-gated field effect transistor (FET) using the as-deposited WS_2_ films on 300 nm thermally grown SiO_2_ on n-type Si substrates (see Fig. S5 for SEM image of the device). Indium was chosen to make direct contact with WS_2_ due to small Schottky barrier which makes it a good choice for ohmic contacts with WS_2_ film^39^. Current voltage measurements were performed in air to evaluate the transfer characteristics of the devices. First, we measured the source-drain current I_ds_ against the voltage between the source and drain V_ds_ for different bottom gate voltages as shown in Fig. 7(A), the linearity of this result reveals that the In/Au electrodes make excellent Ohmic contacts with the WS_2_ channel. To evaluate the transconductance of the device, the back-gate voltage was swept from −91 V to 100 V in both directions as shown in Fig. 7(B). In forward sweep, the device shows n-type behaviour with 6.2 × 10^−5^ cm^2^/V.s field effect mobility, a threshold voltage of −54 V and on/off ratio of 2.5. The field effect mobility was extracted from the slope of the linear part of the transfer curve using the equation:^15^

1 $${\mu }_{FE}=\frac{L}{W{C}_{OX}{V}_{ds}}\frac{\Delta I}{\Delta {V}_{g}}$$

Where μ_FE_ is the field effect mobility, L is the channel length (10 μm), W is the channel width (200 μm), C_ox_ is the capacitance per unit area of silicon dioxide layer (300 nm) which is (11.5 nF/cm^2^), V_ds_ is the source-drain voltage and (ΔI/ΔV_g_) is the transconductance. Interestingly, in backward sweep the FET shows asymmetric ambipolar behaviour with minimum conductivity at 0 V in the n-type branch. However, the low mobility, on-off ratio and the change of the behaviour could be partially attributed to adsorbates from ambient and/or dopants that occur during photolithography and lift-off process. The field effect mobility and the on-off ratio of our WS_2_ FET devices are comparable with backgated WS_2_ FET devices grown by other solution-based approach when they were characterized in air^40^. Additionally, our WS_2_ FET devices show comparable performance with MoS_2_ FET devices fabricated using identical synthesis processes, where the mobility of MoS_2_ devices varied from 10^−4^ to 10^−2^ cm^2^/V.s although the back-gate voltage of these devices was swept to much higher voltages^11,15^. The device field effect characteristics are dictated by the nanocrystalline nature of the film as well as ambient adsorbates. However, as it has been shown with MoS_2_ films that are grown in similar ways, we expect a dramatic improvement when the device is optimized and operated in a top-gate configuration with a high-k dielectric such as HfO_2_^13^ or ionic liquid gate^14,18^.

## Conclusions

In conclusion, we propose a facile and cost-effective growth method that can produce high quality, continuous and ultra-thin WS_2_ films for electronic applications. This solution-based method utilizes thermal decomposition of uniform spin coated ammonium tetrathiotungstate films on two different types of substrates with centimetre scale to create thin WS_2_ films. The key factor that enables (NH_4_)_2_WS_4_ to successfully form a uniform and continuous film is our optimized solvents recipe with volume ratio 3/6 NMP, 2/6 n-butylamine and 1/6 2-aminoethanol that promotes the solubility and wettability of the precursor solution. Different characterization tools were used to confirm the thickness and the quality of the WS_2_ films. Finally, we demonstrated for the first time a back-gated FET from an as-deposited WS_2_ film grown by our solution based process with an electron mobility reaching 6.2 × 10^−5^ cm^2^/V.s which shows comparable performance to MoS_2_ devices fabricated by similar synthesis approaches.

## Materials and Methods

### Preparation of the substrates and (NH_4_)_2_WS_4_ solutions for growth of WS_2_ films

We used 1.5 × 1.5 cm^2^ SiO_2_/Si (300 nm SiO_2_) and sapphire substrates to assess the optimum spin coating conditions for different solvent solutions. Prior to spin coating, the surface of the substrates was cleaned using acetone, isopropanol and de-ionized water followed by conditioning using oxygen plasma at 0.1 mb pressure (oxygen flow 1000 mL/min) and 1000 W power for 15 min to enhance the wettability. To prepare the solutions, we dissolved 174 mg of (NH_4_)_2_WS_4_ in 5 mL of each solvent (DMF, ethylene glycol, NMP) to form 100 mM of precursor solution. After one hour of sonication (at 70 °C) the three solutions were spin coated on the substrates at three different speeds (3000, 6000 and 9000 rpm) for 1 min (step 1: ramp 5 sec, dwell time 5 sec, rpm 500; step 2: ramp 5 sec, dwell time 45 sec, rpm 3000, 6000 and 9000). After spinning, the substrates were prebaked at 140 °C for 1 min using a hot-plate where the solvents evaporated. Prior to thermal decomposition, the refined recipe for the new solvents system we propose (6 mL total volume) is 3/6 NMP, 2/6 butylamine and 1/6 2-aminoethanol. We mixed these solvents together and dissolved (208, 73, 42 and 21) mg of (NH_4_)_2_WS_4_ to create solutions of (100, 35, 20, 10) mM. The solutions were then sonicated for 1 hour at 70 °C before being spin coated at 6000 rpm (the same spin-coating recipe was used as before) on the cleaned and oxygen plasma treated substrates (the same oxygen plasma recipe was used as before). Finally, the samples were baked on a hot-plate at 140 °C for 1 minute.

### Thermal decomposition

The 35 mM concentration samples were used for the thermal decomposition. They were placed in a tube furnace and purged with a 6% H_2_ in Ar gas at 8 mb pressure for 5 minutes. The samples were kept in the cold zone and the furnace was programmed to reach 500 °C. After 20 minutes of temperature stabilization, the samples were moved in the hot zone of the furnace. After 30 min of annealing at 500 °C, the samples were removed from the furnace and were left to cool down naturally while maintaining the flow of gas. To improve the crystallinity of our films we performed a second annealing step at 1000 °C. The samples with identical substrates were arranged in film-facing pairs to prevent the reduction of the films (see Fig. S6). Firstly, the furnace tube was purged with Argon at a pressure of 1 mb for 5 minutes to remove oxygen from the system. The system was then allowed to reach atmospheric pressure under the same 100 sccm Ar flow. After a 40 min temperature ramp, the furnace reached 1000 °C to anneal the samples for 15 minutes before removal from the hot zone to let the samples cool down to room temperature under the same gas and pressure conditions. See Fig. S7 for optical microscopy image of the final WS_2_ film grown on SiO_2_/Si.

### Device fabrication

The transistor channels were formed by conventional photolithography using S1813 photoresist masking and etched for 2 minutes to remove unwanted WS_2_ film regions by Argon ion milling using Oxford Plasma Technology Ionfab 300 plus system. An Argon ion plasma beam was accelerated to 500 V with 100 mA current. The sample placed on a cooled plate (15 °C) at an angle of 45° with respect to the beam and rotated at 5 rpm. The samples were then immersed in acetone to remove the photoresist mask and obtain a WS_2_ channel of 200 μm width which represents the FET. The length of the channel is 10 μm and was defined by the source and drain electrodes positions as patterned by S1805 photoresist. Indium contacts (10 nm thick) were deposited and capped by 50 nm of Au using an e-beam evaporator followed by lift-off.

### Characterization

AFM images were produced using an Agilent 5500 scanning probe microscope. Raman and PL spectroscopy were conducted using Invia Raman Microscope (Renishaw) system with a 532 nm excitation wavelength at 20 mW power and 50X objective. XPS has been performed using a Thermo fisher scientific Thetaprobe system. SEM was performed using a Joel JSM-7500F FEG-SEM. The preparation of the lamella was performed using a Zeiss NVision 40 CrossBeam FIB system. TEM was performed in the Loughborough Materials Characterisation Centre using an FEI Tecnai F20. Electrical measurements were performed in air using an Agilent 4155C semiconductor parameter analyser connected to a cascade micropositioning stage.

## Supplementary information

Supplementary Information.

## References

[1] Fiori G (2014). Electronics based on two-dimensional materials. Nat. Nanotechnol..

[2] Chhowalla M (2013). The chemistry of two-dimensional layered transition metal dichalcogenide nanosheets. Nat. Chem..

[3] Mak KF, Lee C, Hone J, Shan J, Heinz TF (2010). Atomically thin MoS_2_: A new direct-gap semiconductor. Phys. Rev. Lett..

[4] Splendiani A (2010). Emerging photoluminescence in monolayer MoS_2_. Nano Lett..

[5] Mak KF, McGill KL, Park J, McEuen PL (2014). Valleytronics. The valley Hall effect in MoS_2_ transistors. Science.

[6] Radisavljevic B, Radenovic A, Brivio J, Giacometti V, Kis A (2011). Single-layer MoS_2_ transistors. Nat. Nanotechnol..

[7] Coleman JN (2011). Two-dimensional nanosheets produced by liquid exfoliation of layered materials. Science.

[8] Tan LK (2014). Atomic layer deposition of a MoS_2_ film. Nanoscale.

[9] Amani M (2016). High Luminescence Efficiency in MoS_2_ Grown by Chemical Vapor Deposition. ACS Nano.

[10] Samadi M (2018). Group 6 transition metal dichalcogenide nanomaterials: Synthesis, applications and future perspectives. Nanoscale Horizons.

[11] Liu K-K (2012). Growth of Large-Area and Highly Crystalline MoS_2_ Thin Layers on Insulating Substrates. Nano Lett..

[12] George AS (2014). Wafer Scale Synthesis and High Resolution Structural Characterization of Atomically Thin MoS_2_ Layers. Adv. Funct. Mater..

[13] Yang J (2015). Wafer-scale synthesis of thickness-controllable MoS_2_ films via solution-processing using a dimethylformamide/n-butylamine/2-aminoethanol solvent system. Nanoscale.

[14] Lim YR (2016). Wafer-Scale, Homogeneous MoS_2_ Layers on Plastic Substrates for Flexible Visible-Light Photodetectors. Adv. Mater..

[15] Hung YH (2016). Scalable Patterning of MoS_2_ Nanoribbons by Micromolding in Capillaries. ACS Appl. Mater. Interfaces.

[16] Ionescu R (2017). Chelant Enhanced Solution Processing for Wafer Scale Synthesis of Transition Metal Dichalcogenide Thin Films. Sci. Rep..

[17] Yang H (2017). Highly Scalable Synthesis of MoS_2_Thin Films with Precise Thickness Control via Polymer-Assisted Deposition. Chem. Mater..

[18] Lim YR (2018). Roll-to-Roll Production of Layer-Controlled Molybdenum Disulfide: A Platform for 2D Semiconductor-Based Industrial Applications. Adv. Mater..

[19] Zhao W (2013). Evolution of Electronic Structure in Atomically Thin Sheets of WS_2_ and WSe_2_. ACS Nano.

[20] Liu L, Kumar SB, Ouyang Y, Guo J (2011). Performance limits of monolayer transition metal dichalcogenide transistors. IEEE Trans. Electron Devices.

[21] Hwang WS (2012). Transistors with chemically synthesized layered semiconductor WS_2_ exhibiting 10^5^ room temperature modulation and ambipolar behavior. Appl. Phys. Lett..

[22] Orofeo CM, Suzuki S, Sekine Y, Hibino H (2014). Scalable synthesis of layer-controlled WS_2_ and MoS_2_ sheets by sulfurization of thin metal films. Appl. Phys. Lett..

[23] Gutiérrez HR (2013). Extraordinary room-temperature photoluminescence in triangular WS_2_ monolayers. Nano Lett..

[24] Kwon KC (2017). Tungsten disulfide thin film/p-type Si heterojunction photocathode for efficient photochemical hydrogen production. MRS Commun..

[25] Li Z (2016). Facile synthesis of large-area and highly crystalline WS_2_ film on dielectric surfaces for SERS. J. Alloys Compd..

[26] Kwon KC (2015). Synthesis of atomically thin transition metal disulfides for charge transport layers in optoelectronic devices. ACS Nano.

[27] Annamalai M (2016). Surface energy and wettability of van der Waals structures. Nanoscale.

[28] Srinivasan BR, Näther C, Dhuri SN, Bensch W (2006). On the importance of H-bonding interactions in organic ammonium tetrathiotungstates. Monatshefte fur Chemie.

[29] Srinivasan BR, Naik AR, Näther C, Bensch W (2007). Synthesis, spectroscopy and crystal structures of chiral organic ammonium tetrathiometalates showing N-H⋯S and C-H⋯S interactions. Zeitschrift fur Anorg. und Allg. Chemie.

[30] Zhao W (2013). Lattice dynamics in mono- and few-layer sheets of WS_2_ and WSe_2_. Nanoscale.

[31] Bissett MA, Hattle AG, Marsden AJ, Kinloch IA, Dryfe RAW (2017). Enhanced Photoluminescence of Solution-Exfoliated Transition Metal Dichalcogenides by Laser Etching. ACS Omega.

[32] Su L, Yu Y, Cao L, Zhang Y (2015). Effects of substrate type and material-substrate bonding on high-temperature behavior of monolayer WS_2_. Nano Res..

[33] Berkdemir A (2013). Identification of individual and few layers of WS_2_ using Raman Spectroscopy. Sci. Rep..

[34] Li DH (2017). Dielectric functions and critical points of crystalline WS_2_ ultrathin films with tunable thickness. Phys. Chem. Chem. Phys..

[35] Lan F (2018). Synthesis of large-scale single-crystalline monolayer WS_2_ using a semi-sealed method. Nanomaterials.

[36] Sang Y (2015). From UV to near-infrared, WS_2_ nanosheet: A novel photocatalyst for full solar light spectrum photodegradation. Adv. Mater..

[37] Tan SM, Pumera M (2016). Bottom-up Electrosynthesis of Highly Active Tungsten Sulfide (WS_3-x_) Films for Hydrogen Evolution. ACS Appl. Mater. Interfaces.

[38] Alsabban MM (2016). Editors’ Choice Growth of Layered WS_2_ Electrocatalysts for Highly Efficient Hydrogen Production Reaction. ECS J. Solid State Sci. Technol..

[39] Wang Y (2019). Van der Waals contacts between three-dimensional metals and two-dimensional semiconductors. Nature.

[40] Lan C, Li C, Yin Y, Liu Y (2015). Large-area synthesis of monolayer WS_2_ and its ambient-sensitive photo-detecting performance. Nanoscale.

