# Supplementary material for: Solution-Based Synthesis of Few-Layer WS2 Large Area Continuous Films for Electronic Applications
Source: Sci Rep. 2020 Feb 3;10:1696. doi: 10.1038/s41598-020-58694-0 (PMC6997350; doi:10.1038/s41598-020-58694-0)
Supplement: Supplementary file 1 — Supplementary Information. [file 41598_2020_58694_MOESM1_ESM.docx]

Supplementary Information

Solution-Based Synthesis of Few-Layer WS_2_ Large Area Continuous Films for Electronic Applications

Omar A. Abbas^1†^, Ioannis Zeimpekis^1†^, He Wang^2^, Adam H. Lewis^1^, Neil P. Sessions^1^, Martin Ebert^3^, Nikolaos Aspiotis^1^, Chung-Che Huang^1^, Daniel Hewak^1^, Sakellaris Mailis^1,4^ & Pier Sazio^1*^ ‎

^1^Optoelectronics Research Centre, University of Southampton, Southampton, SO17 1BJ, United Kingdom

^2^National Centre for Advanced Tribology, University of Southampton, Southampton, SO17 1BJ, United Kingdom

^3^School of Electronics and Computer Science, University of Southampton, Southampton, SO17 1BJ, United Kingdom

**Present address:** ^4^Skolkovo Institute of Science and Technology Novaya St., 100, Skolkovo 143025, Russian Federation

^†^These authors contributed equally to this work.

**Corresponding Author:** Pier Sazio

^*^ E-mail: [pjas@soton.ac.uk](mailto:pjas@soton.ac.uk)


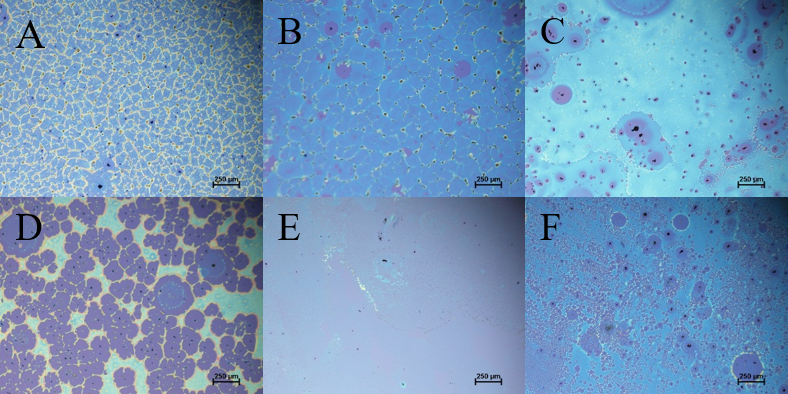


**Figure S1**. Optical microscope images of precursor films prepared by dissolving 100 mM of (NH_4_)_2_WS_4_ in: dimethylformamide (DMF) solvent then spun coated at (A) 3000 and (D) 9000 rpm spinning speed respectively; ethylene glycol solvent then spun coated at (B) 3000 and (E) 9000 rpm respectively; n-methylpyrrolidone (NMP) solvent then spun coated at (C) 3000 and (F) 9000 rpm respectively.


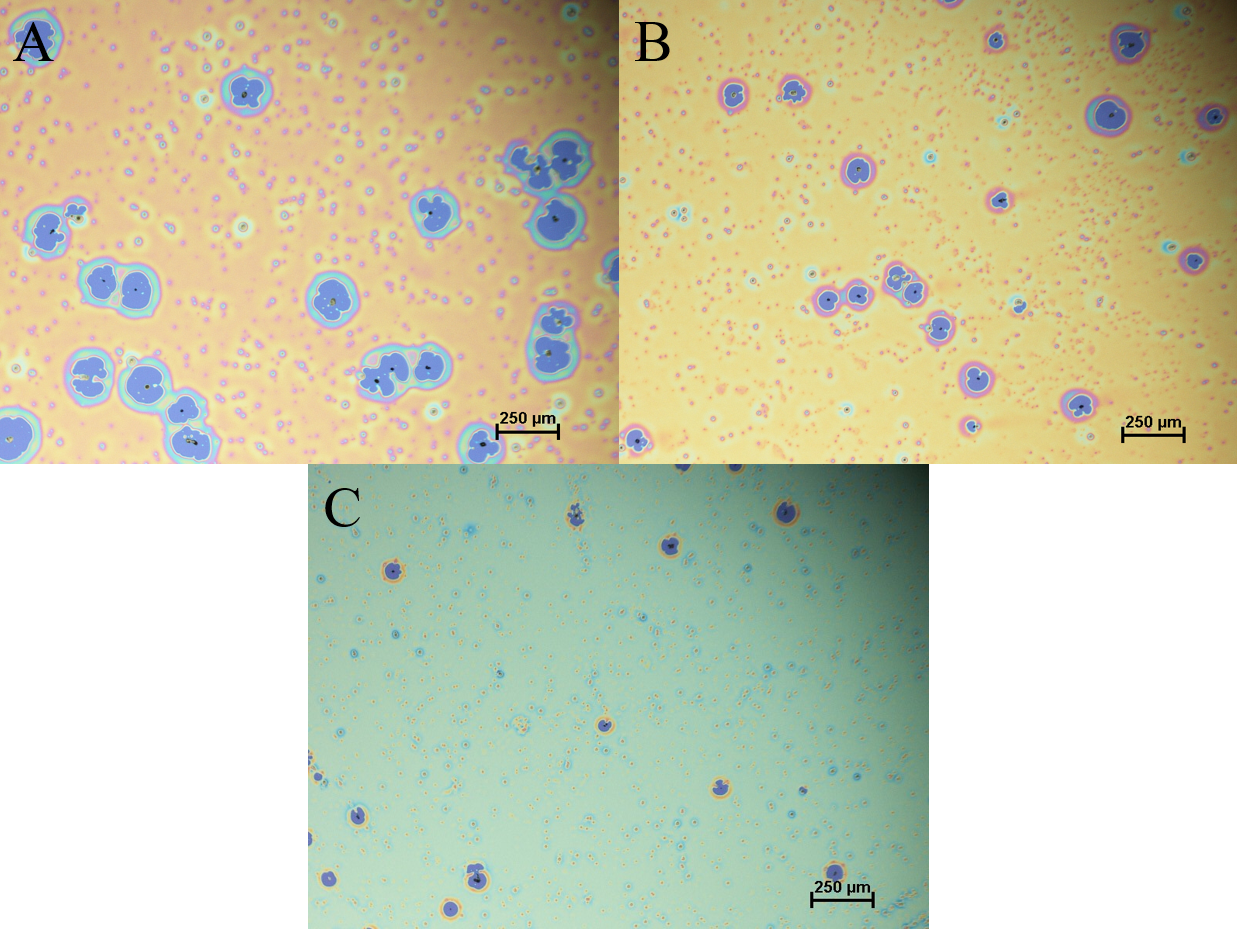


**Figure S2**. Optical microscope images of precursor films prepared by dissolving 100 mM of (NH_4_)_2_WS_4_ in 5 mL of (2/5 dimethylformamide (DMF), 2/5 n-butylamine 1/5 2-aminoethanol) then spun coated at: (A) 3000, (B) 6000 and (C) 9000 rpm. This solvent recipe is a modified recipe from ref (13) which can create highly uniform (NH_4_)_2_MoS_4_ films by spin-coating that can thermally decompose to produce MoS_2_ films. However, when this recipe was applied to create (NH_4_)_2_WS_4_ films, large size pinholes (≥50µm) exist in addition to undissolved (NH_4_)_2_WS_4_ particles.


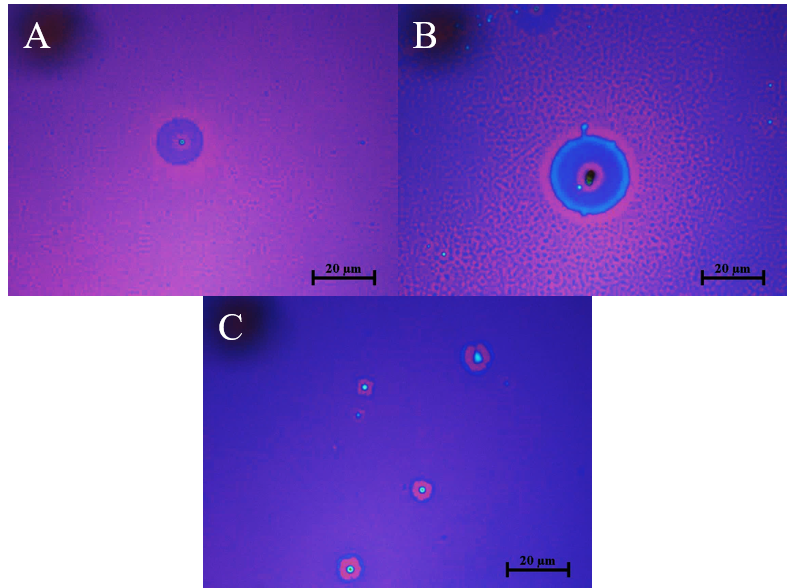


**Figure S3**. Optical microscope images of spin-coated precursor films prepared by dissolving (NH_4_)_2_WS_4_ in our solvent recipe which is 6 mL of (3 mL NMP, 2 mL butylamine and 1 mL 2-aminoethanol) using different concentrations (spin coating at 6000 rpm for 1 min). (A) 10 mM concentration: the film is not continuous and contains low density of precursor islands. (B) 20 mM concentration: the film has higher density of precursor islands but still not continuous. (C) 35 mM film is the minimum concentration to create uniform and continuous (NH_4_)_2_WS_4_ ‎films. However, few micron-size defects (≤10µm) occurred in this primary sample due to debris or insoluble particles of the precursor which adhered on the surface of the substrates. Almost defect-free precursor films have been obtained (see Figure 1(F)) after further optimisation in the sonication process of precursor solution and cleaning procedure of the substrates.


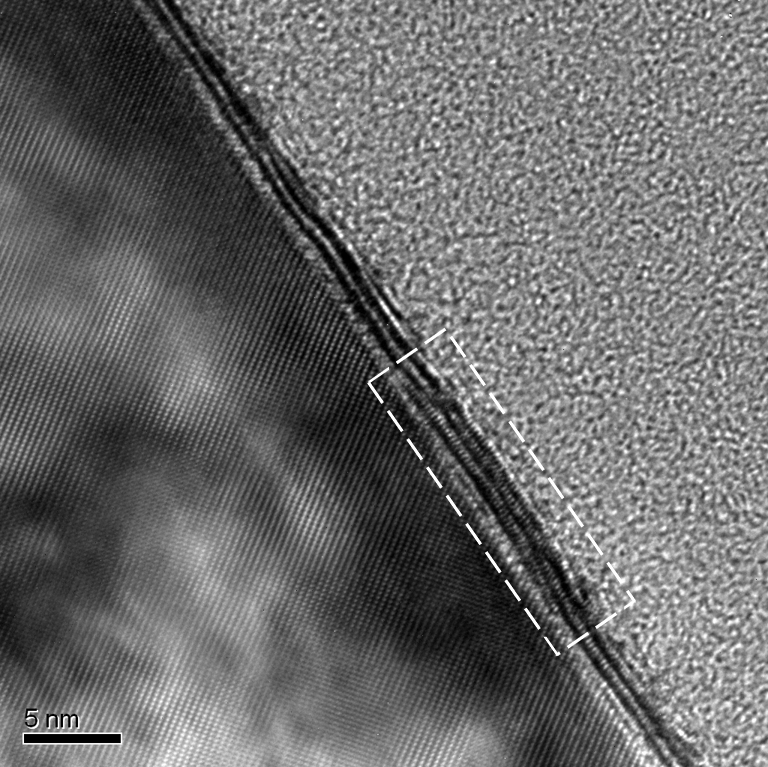


**Figure S4**. TEM image of few-layer WS_2_ films grown on sapphire substrate. The crystalline Al_2_O_3_ atomic lattice is ‎clearly visible on the left-hand side of the image. The WS_2_ film is viewed at a high angle where bilayer and ‎trilayer regions are also highly visible. The bright area on the right-hand side is the protective carbon coating. The cross-section of few-layer WS_2_ film shows that the atomic columns are clearly visible confirming the crystallinity order of the film as indicated by dotted box.

It is worth to mention that the TEM lamella was prepared using a Zeiss FIB. The thinning process was performed using a gallium ion beam and introduced some damage in some areas. The result of this damage is that the atoms in certain areas were not resolved by the TEM. However, there are a few undamaged areas where the atom columns are clearly discernible as shown in Fig. S4.


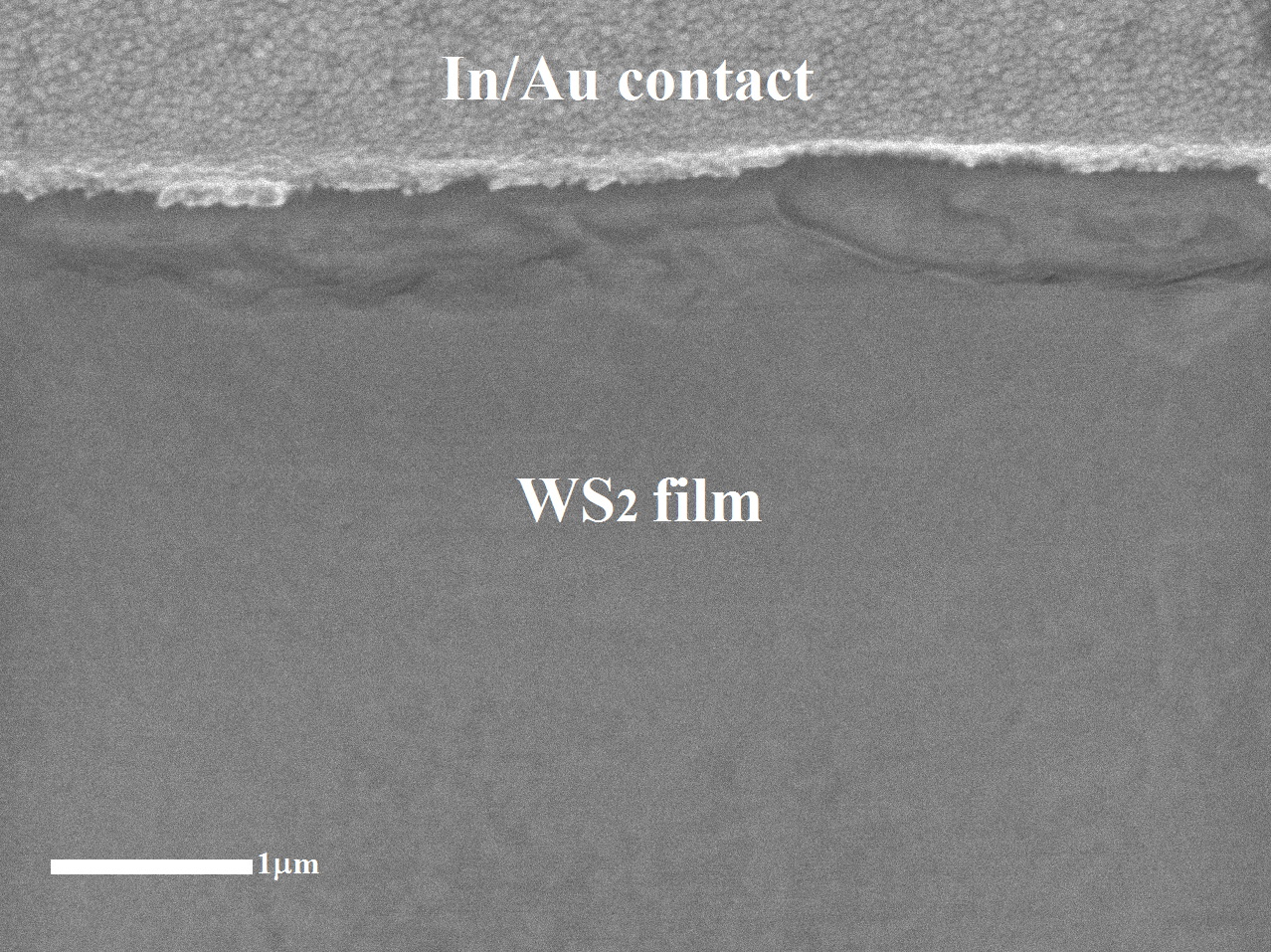
**Figure S5**. Scanning electron microscopy (SEM) image for a section of few-layer WS_2_ FET device which shows the In/Au contact and WS_2_ film grown on SiO_2_/Si substrate. As can be seen from this SEM image, the WS_2_ film is homogenous and featureless due to its high uniformity and is continuous without any discernible defects or pinholes. The darker areas at the interface between the contact and the film and over the film are caused by the residue of the photoresist that occurred due to imperfect lift-off process.


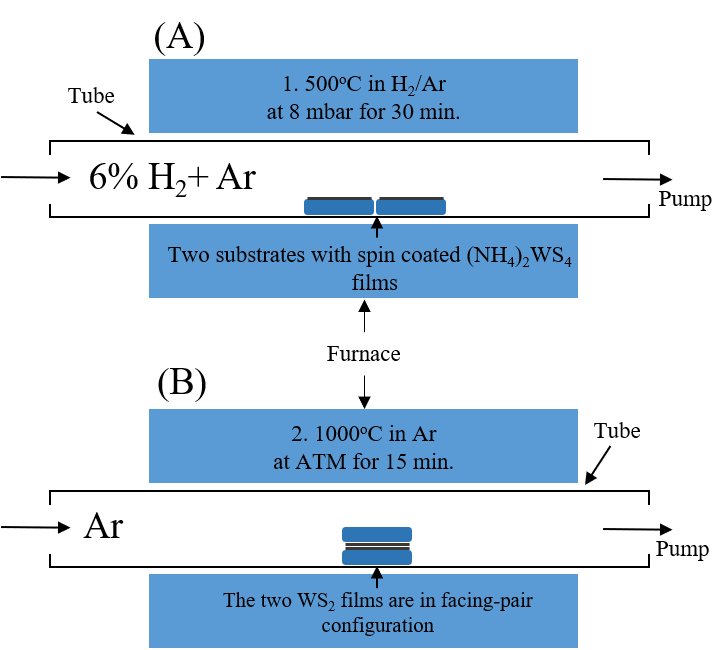


**Figure S6**. Schematic diagram of WS_2_ solution-based synthesis by two annealing steps where (A) is the first annealing step to thermally decompose the spin coated (NH_4_)_2_WS_4_ films and (B) is the second annealing step to improve the crystallinity of the WS_2_ film.


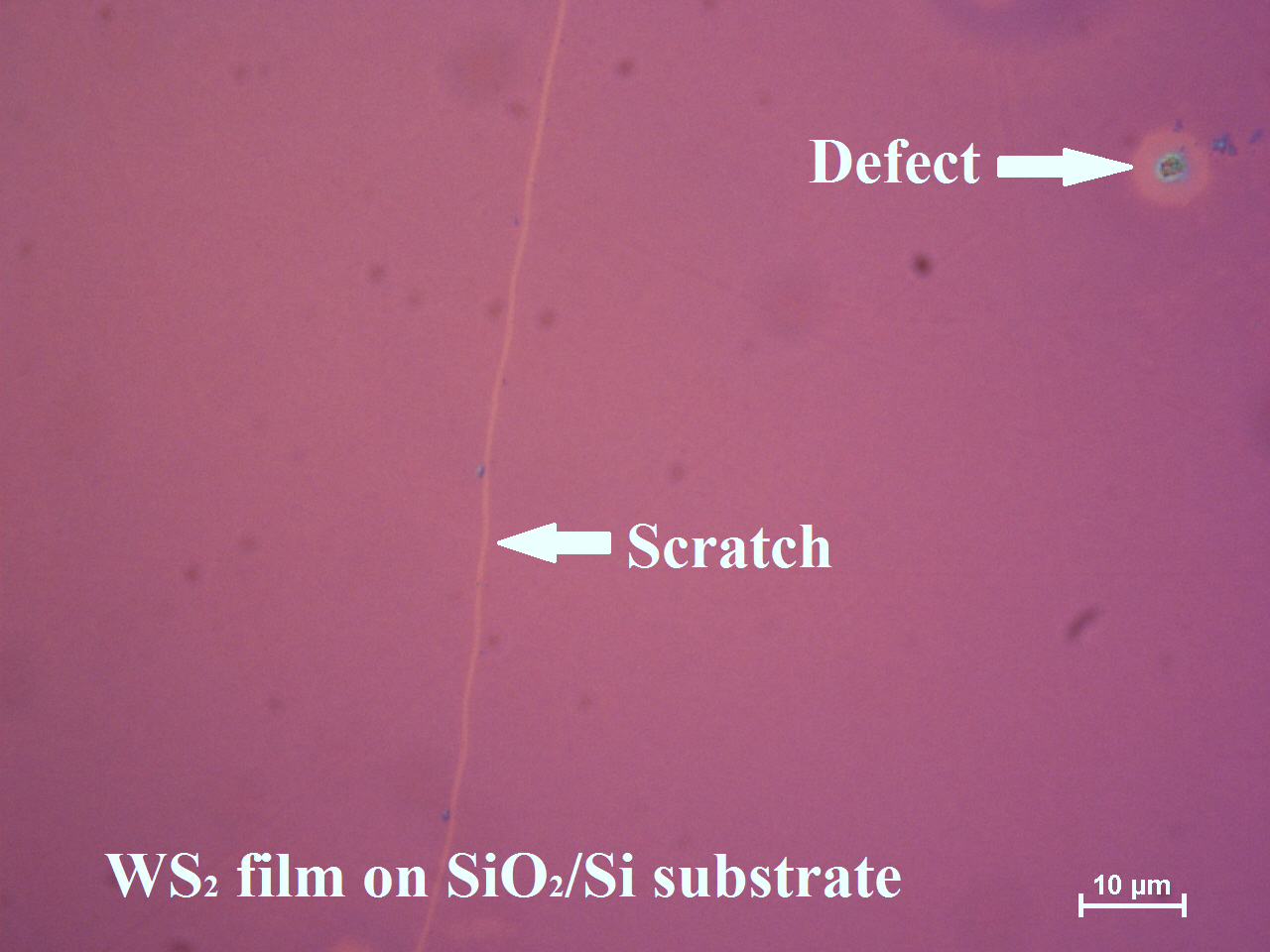


**Figure S7**. Optical microscope image of few-layer WS_2_ film grown on SiO_2_/Si substrate by two-step thermal decomposition using precursor concentration 35 mM dissolved in our solvent recipe.
